# Expanding the role of ultrasonography in cardiopulmonary assessment in dromedary camels

**DOI:** 10.3389/fvets.2025.1671030

**Published:** 2025-09-25

**Authors:** Mohamed Tharwat

**Affiliations:** Department of Clinical Sciences, College of Veterinary Medicine, Qassim University, Buraidah, Saudi Arabia

**Keywords:** camels, diseases, heart, lung, ultrasonography

## Abstract

Dromedary camels (*Camelus dromedarius*) have unique cardiopulmonary anatomy and physiology that pose diagnostic challenges unlike those in other large ruminants and equids. Ultrasonography, as a non-invasive, portable, and cost-effective imaging tool, offers great potential for cardiopulmonary evaluation—especially in field conditions where early detection is crucial. This review article explores the expanding role of ultrasonography in assessing the camel heart and lungs, emphasizing recent advances and key clinical applications. It begins by outlining anatomical and physiological features relevant to ultrasound imaging, including cardiac chambers, valves, and pulmonary structures. Principles of ultrasound physics, equipment choices, scanning techniques, and practical considerations such as animal restraint and probe placement are detailed. Normal ultrasonographic findings are summarized to establish baseline reference values. The review highlights the utility of ultrasonography in diagnosing a range of conditions, including pneumonia, pulmonary abscesses, pneumothorax, pulmonary emphysema, pleural effusion, pleurisy, pleuropneumonia, pericardial effusion, endocarditis, pericarditis, myocardial degeneration, as well as calcified and hypertrophic cardiomyopathy. Comparisons with radiography and CT illustrate ultrasound’s strengths in real-time, bedside diagnosis. Notably, the review identifies a critical gap: the lack of standardized protocols and normative datasets for camelids. Addressing these limitations through clinician training, species-specific guidelines, and research into advanced techniques such as Doppler and contrast-enhanced ultrasonography is essential. This review underscores the need for innovation and collaboration to optimize ultrasonographic diagnostics in camelid cardiopulmonary care.

## 1 Introduction

Dromedary camels (*Camelus dromedarius*) play a pivotal role in sustaining livelihoods and ecosystems across arid and semi-arid regions, particularly in North Africa, the Middle East, and parts of South Asia (1). Their unique physiological adaptations allow them to thrive in harsh climates where other livestock species struggle, contributing significantly to food security, transport, and income generation (2, 3). In addition to their resilience to climate change and low water dependency, camels have growing importance in commercial sectors such as milk production and racing (4). Understanding camel health and performance, particularly in relation to cardiopulmonary function, is therefore critical not only for animal welfare but also for the socioeconomic and ecological stability of the regions that depend on them (5, 6).

Cardiopulmonary disorders in dromedary camels, though historically underdiagnosed, are increasingly recognized as important contributors to morbidity, decreased productivity, and economic loss in both traditional and commercial camel-rearing systems (7). Respiratory conditions such as pneumonia, pleuritis, and bronchopneumonia are commonly reported, especially in young animals and during seasonal weather changes, often linked to bacterial, viral, or parasitic infections (8). Cardiovascular abnormalities, including hypertrophic cardiomyopathy, congestive heart failure, pericarditis, myocarditis, and valvular defects, although less frequently described in the literature, are emerging as notable concerns, particularly with improvements in diagnostic capabilities (9–17). Additionally, environmental stressors such as heat, dust exposure, and long-distance transport have been associated with subclinical respiratory compromise, which can affect athletic and working performance (18). Despite the growing awareness of these conditions, comprehensive epidemiological data remain scarce, partly due to limited access to advanced diagnostic tools in the field (14). However, early and accurate diagnosis remains challenging due to the nonspecific presentation of many cardiopulmonary conditions and the limited availability of accessible, species-specific diagnostic tools (14, 19).

The diagnosis of cardiopulmonary diseases in dromedary camels presents unique challenges due to a combination of anatomical, behavioral, and logistical factors (7). Unlike more commonly studied domestic species, camels possess a distinctive thoracic anatomy, including a relatively deep and narrow chest cavity and a high resistance to overt clinical expression of disease, which complicates both auscultation and physical examination findings (18). Their stoic temperament often masks early signs of illness, leading to late-stage clinical presentation and delayed intervention (19, 20). Moreover, the scarcity of species-specific diagnostic reference values and validated protocols further limits the utility of standard veterinary tools such as radiography and electrocardiography, which are often adapted from cattle or equine models (21). Field conditions in camel-rearing regions can also restrict access to sophisticated diagnostic equipment, and the large size of adult camels poses practical difficulties for imaging and restraint, particularly in non-sedated individuals (22). These constraints underscore the importance of implementing non-invasive, portable, and species-adapted diagnostic modalities to improve early detection and clinical management.

Ultrasonography has emerged as a valuable diagnostic modality in veterinary medicine due to its non-invasive nature, real-time imaging capability, and portability—attributes that make it especially suitable for use in large animal species such as the dromedary camel (10, 23–30). Unlike radiography, which is limited by the camel’s size and thoracic depth, ultrasound allows for dynamic visualization of both cardiac and pulmonary structures without exposure to ionizing radiation (31). The ability to perform bedside imaging in field conditions is particularly important in camelid practice, where transporting large animals to specialized facilities is often impractical. Ultrasonography also facilitates early detection of thoracic abnormalities such as pleural effusion, pericardial fluid accumulation, lung consolidation, and cardiac chamber enlargement, which may otherwise remain undiagnosed until clinical deterioration occurs (7). Furthermore, advancements in transducer technology and image resolution have enhanced the diagnostic utility of thoracic ultrasonography, enabling clinicians to distinguish between various pulmonary and cardiac pathologies with increasing precision (32). When compared to cattle and horses, ultrasonography in camels demonstrates comparable or even superior diagnostic sensitivity for certain thoracic conditions, particularly due to the camel’s relatively less aerated lung field and more accessible thoracic window. Given these advantages, ultrasonography offers a promising solution to many of the diagnostic challenges faced in camel medicine, yet its use remains underexplored and inconsistently standardized.

Despite increasing recognition of the clinical and economic significance of cardiopulmonary diseases in dromedary camels, diagnostic advancements have lagged behind those in other large animal species (31). Ultrasonography has shown considerable promise as a safe, accessible, and field-adaptable imaging modality for thoracic assessment; however, its application in camels remains underexplored and inconsistently standardized (32). The primary objective of this review is to synthesize and critically evaluate the existing literature on the use of ultrasonography in the cardiopulmonary evaluation of dromedary camels, highlighting both its diagnostic capabilities and limitations. This review aims to bridge current knowledge gaps by outlining normal thoracic sonographic anatomy, identifying key ultrasonographic findings associated with common cardiac and pulmonary disorders, and discussing recent technological innovations that may enhance field-based diagnostics. Furthermore, it seeks to propose future research directions and clinical applications that could support the integration of ultrasonography into routine veterinary practice in camelid medicine. By consolidating existing data and offering a forward-looking perspective, this review intends to facilitate improved diagnostic precision, animal welfare, and disease management strategies in camel health systems.

## 2 Anatomy and physiology of the camel cardiopulmonary system

### 2.1 Unique anatomical and physiological features of dromedary camels

**Figure 1:**
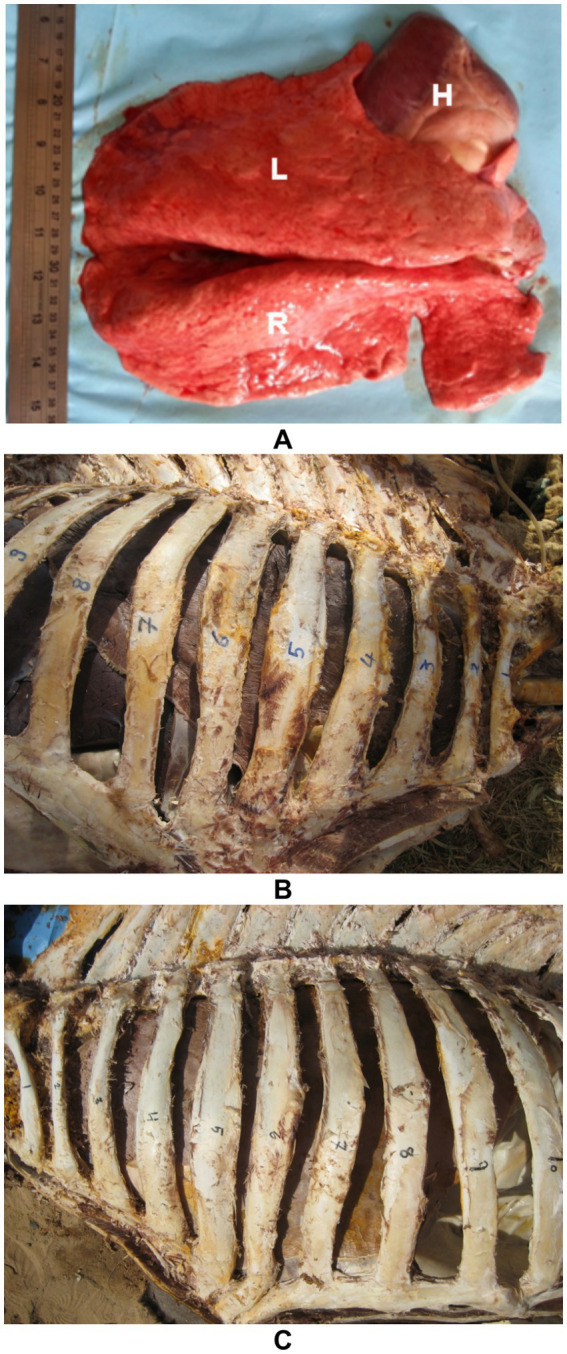
Topographic and gross anatomy of the lungs in dromedary camels. **(A)** Postmortem image showing a healthy camel right (R) and left (L) lungs, and heart (H) *in situ*. **(B)** Right and **(C)** left thoracic cavities of a formalin-preserved camel cadaver illustrating lung topography. Rib numbers are indicated to demonstrate lung positioning relative to the thoracic skeleton. These views aid in understanding anatomical landmarks for ultrasound-guided thoracic examination [modified from Tharwat and Al-Sobayil (34)].

Dromedary camels possess a range of distinctive anatomical and physiological adaptations that support survival in arid and extreme environments, many of which influence the structure and function of the cardiopulmonary system (18). Anatomically, the thorax is narrow at the cranial end but widens significantly toward the caudal end due to the outward curvature of the costal arches. The lungs are soft, spongy, and notably lack fissures. Each lung is divided into cranial and caudal lobes, with the right lung also featuring an accessory lobe (33). Although lobulation is not prominent, small lobules separated by connective tissue can be observed upon closer examination (34) (Figure 1). The thoracic cavity of the camel is deep and laterally compressed, with well-developed intercostal musculature and a thick dermal layer, which collectively present both a protective advantage and a challenge for imaging modalities such as ultrasonography (18). The lungs are relatively small in proportion to body size but exhibit high compliance and efficiency, contributing to effective oxygen exchange even under heat stress and dehydration (35, 36). Additionally, the pulmonary vasculature is capable of significant constriction and dilation, enabling camels to regulate perfusion in response to fluctuating environmental conditions (37). Cardiac adaptations include a relatively large right ventricle and strong myocardial mass, facilitating sustained circulation during prolonged periods of physical exertion or fluid restriction (38). Unlike ruminants, camels exhibit a unique ability to maintain cardiac output and stable blood pressure despite considerable dehydration, attributed to enhanced plasma volume retention and reduced capillary permeability (39).

**Figure 2:**
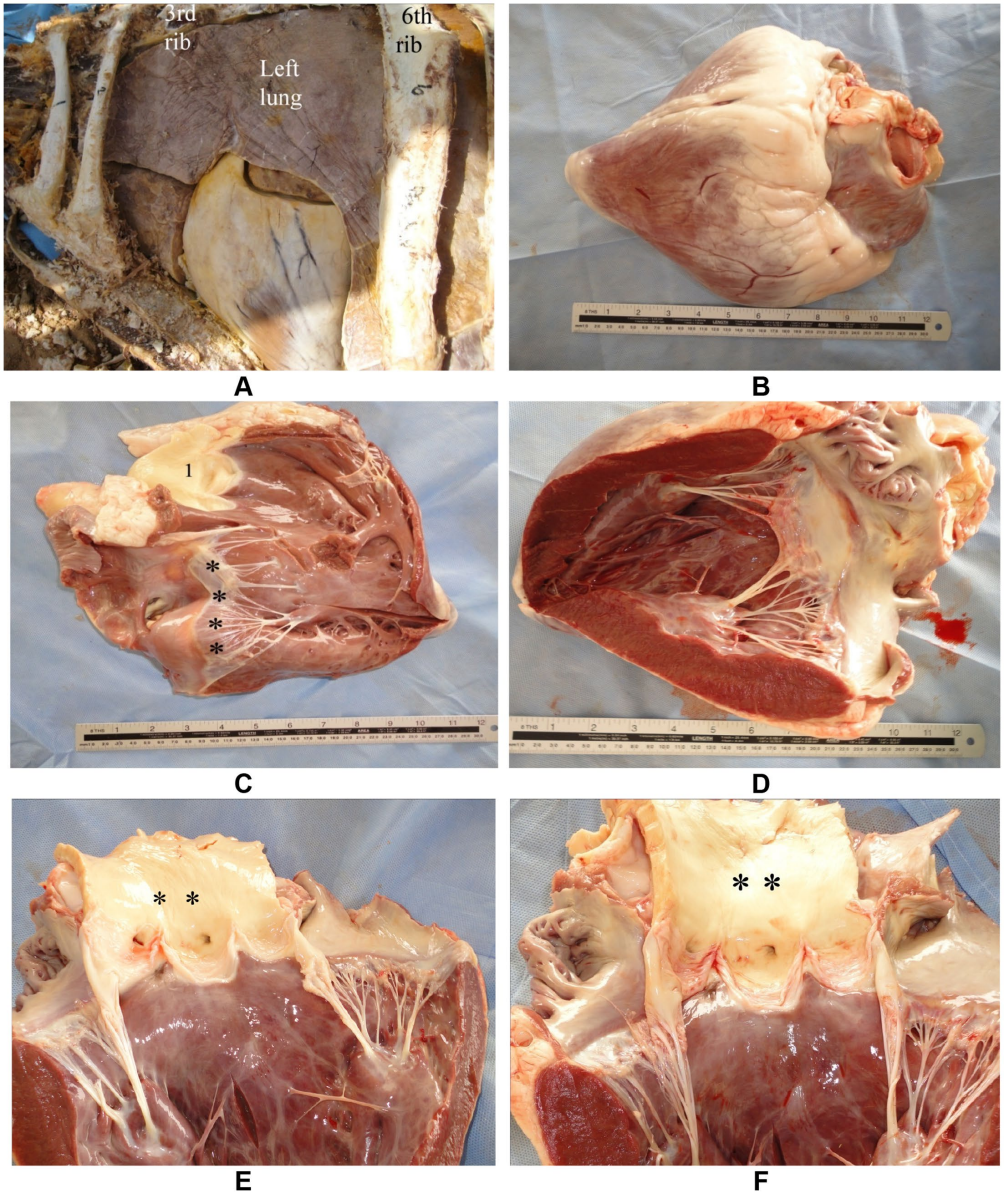
Anatomy of the camel heart in gross dissection. **(A)** Thoracic cavity view showing the position of the heart in a formalin-fixed specimen. **(B)** External heart with a prominent fat-filled coronary groove separating atria from ventricles. **(C)** Tricuspid valve (black asterisks) and pulmonary trunk (1) in situ. **(D)** Mitral valve with clearly visible chordae tendineae anchored to papillary muscles. **(E,F)** Semilunar valves (black asterisks) located at the base of the pulmonary artery and aorta, respectively. These views provide structural orientation for echocardiographic correlation [modified from Tharwat and Al-Sobayil (34)].

The adult camel heart weighs approximately 4–6 kg, accounting for about 0.7% of the body weight. Anatomically, it is located between the 3rd and 6th ribs (specifically the 3rd to 5th intercostal spaces) and lies in contact with the ventral third of the chest wall (33). The deep, fat-filled coronary groove separates the atria from the ventricles. The right and left atria are divided by the interatrial septum. The right atrioventricular (tricuspid) valve encircles the right atrioventricular opening, with its free edges anchored to papillary muscles via tendinous cords known as chordae tendineae. Similarly, the left atrioventricular (mitral) valve surrounds the left atrioventricular opening, with its valvelets also connected to papillary muscles by chordae tendineae (33). The semilunar valves are located at the origins of the pulmonary trunk and the aorta. The pulmonary trunk bifurcates into the left and right pulmonary arteries, which supply the respective lungs. The aorta exits the heart as the descending aorta and, just distal to the semilunar valves, gives rise to two major arteries (34) (Figure 2). These species-specific features not only underline the camel’s resilience but also necessitate tailored approaches in diagnostic imaging and clinical interpretation, particularly in cardiopulmonary evaluation.

### 2.2 Comparative aspects with other large ruminants or equids

While dromedary camels share some traits with large ruminants and equids, their cardiopulmonary system has key differences affecting clinical diagnosis. Camels have a more conical thorax, influencing heart and lung orientation, with a relatively larger, more caudally positioned heart that supports efficient output during exertion or dehydration (18, 38). Their lungs are less segmented than ruminants’, impacting lesion distribution and ultrasound detection (40, 41). Camel erythrocytes are oval and non-nucleated, enabling better blood flow and tolerance to osmotic stress, unlike bovines or horses (42). Physiologically, camels maintain stable cardiopulmonary function under extreme heat and dehydration, unlike cattle and horses (39). Additionally, camels present unique ultrasonographic challenges due to their thick thoracic wall and dense musculature, which can attenuate ultrasound waves and reduce image clarity compared to cattle and horses. This requires the use of lower-frequency probes and adapted scanning techniques to achieve adequate penetration and resolution. Furthermore, the limited intercostal spaces and their narrow spacing can restrict probe maneuverability, making standard cardiac imaging planes more difficult to obtain in camels than in other large domestic species. These differences highlight the need for camel-specific diagnostic standards, as applying data from other species risks inaccuracies.

### 2.3 Relevance to ultrasound imaging

Understanding the unique cardiopulmonary anatomy and physiology of dromedary camels is crucial for accurate ultrasound interpretation and optimized scanning protocols. Their deep thorax, strong muscles, and thick skin require specific probe selection and positioning to image deep cardiac structures and pleural recesses (11). The heart’s caudal, vertical orientation demands tailored parasternal windows for echocardiography (41). Simplified lung lobation and extended pleura necessitate systematic intercostal scanning to detect peripheral lesions (7). Camels’ ability to tolerate dehydration without clear hemodynamic changes calls for dynamic ultrasound methods like M-mode and Doppler for early detection of distress (43, 44). Applying this species-specific knowledge improves diagnostic accuracy and clinical decision-making.

## 3 Principles and techniques of ultrasonography

### 3.1 Basics of ultrasound physics relevant to cardiopulmonary evaluation

Understanding the fundamental principles of ultrasound physics is essential for optimizing its diagnostic use in cardiopulmonary imaging, particularly in large animals such as dromedary camels. Ultrasonography relies on the transmission of high-frequency sound waves—typically between 2 and 15 MHz—into biological tissues, where differences in acoustic impedance between structures generate echoes that are processed into real-time images (45). For thoracic evaluation, lower-frequency transducers (e.g., 3.5–5 MHz) are commonly used to achieve deeper penetration necessary for visualizing cardiac chambers and pulmonary surfaces, though at the expense of image resolution (46). The lung, due to its high air content, normally reflects most of the ultrasound waves, creating characteristic reverberation artifacts such as A-lines and mirror images; however, pathological changes like consolidation, pleural effusion, or abscess formation alter this acoustic interface, enabling meaningful interpretation (47). In cardiac imaging, real-time B-mode and M-mode techniques are employed to assess chamber size, wall motion, and valve function, while Doppler modalities provide insight into blood flow dynamics and intracardiac pressures (48). A firm grasp of these physical principles enhances the clinician’s ability to distinguish normal anatomical patterns from disease-related changes, thereby improving diagnostic accuracy and clinical decision-making in camelid cardiopulmonary evaluations (32).

### 3.2 Equipment and probes typically used in camels

Selecting appropriate ultrasound equipment and transducers is critical to achieving diagnostic-quality images in camelid thoracic assessments, given the animal’s size, thick skin, and unique anatomical features (31). Portable, battery-operated ultrasound units with robust image processing capabilities are often preferred in field settings due to their mobility and ease of use in remote regions where camels are typically examined (49). Transducer selection is largely dictated by the depth and type of tissue being evaluated. For cardiac imaging, phased-array or microconvex transducers operating at frequencies between 2.5 and 5 MHz are generally recommended, as they provide adequate penetration through the thick thoracic wall and allow for sector-shaped views suitable for echocardiography (32). Linear or convex probes with slightly higher frequencies (5–7.5 MHz) are often employed for examining superficial thoracic structures such as the pleura, peripheral lung zones, and the cranial thoracic cavity, especially in younger or thinner animals (7). Adequate skin preparation—typically involving shaving, cleaning, and generous application of acoustic coupling gel—is essential to minimize acoustic impedance and improve image clarity, particularly in adult camels with coarse hair and thick dermis (8).

### 3.3 Techniques for thoracic and cardiac ultrasound in camels

Performing thoracic and cardiac ultrasonography in dromedary camels requires an adapted approach that considers their large size, anatomical conformation, and temperament. For thoracic imaging, the camel is typically examined in a sitting position with minimal restraint (7). A systematic scanning protocol is followed, beginning cranially near the thoracic inlet and progressing caudally along the intercostal spaces to the level of the diaphragm. The transducer is placed parallel to the ribs to minimize acoustic shadowing, with emphasis on the 3rd to 8th intercostal spaces for visualizing pleural surfaces, lung fields, and peripheral lesions such as consolidation, abscesses, or pleural effusions (8). Cardiac ultrasonography is performed from the right and left parasternal windows, ideally using a phased-array or microconvex transducer. The right parasternal long-axis view is commonly used to evaluate cardiac chambers, wall thickness, and valve motion, while short-axis views are useful for cross-sectional assessment of ventricular function and outflow tracts (11, 50). M-mode imaging allows for quantitative measurements of myocardial contractility, and Doppler modalities—when available—enable the evaluation of intracardiac flow patterns and valvular insufficiencies (43). Proper probe orientation, consistent anatomical landmarks, and species-specific adjustments in scanning depth and angle are critical for obtaining interpretable and repeatable images in camels, where thoracic structures lie deeper than in other domestic animals due to the thick body wall and unique thoracic configuration (32).

### 3.4 Positioning and restraint

**Figure 3:**
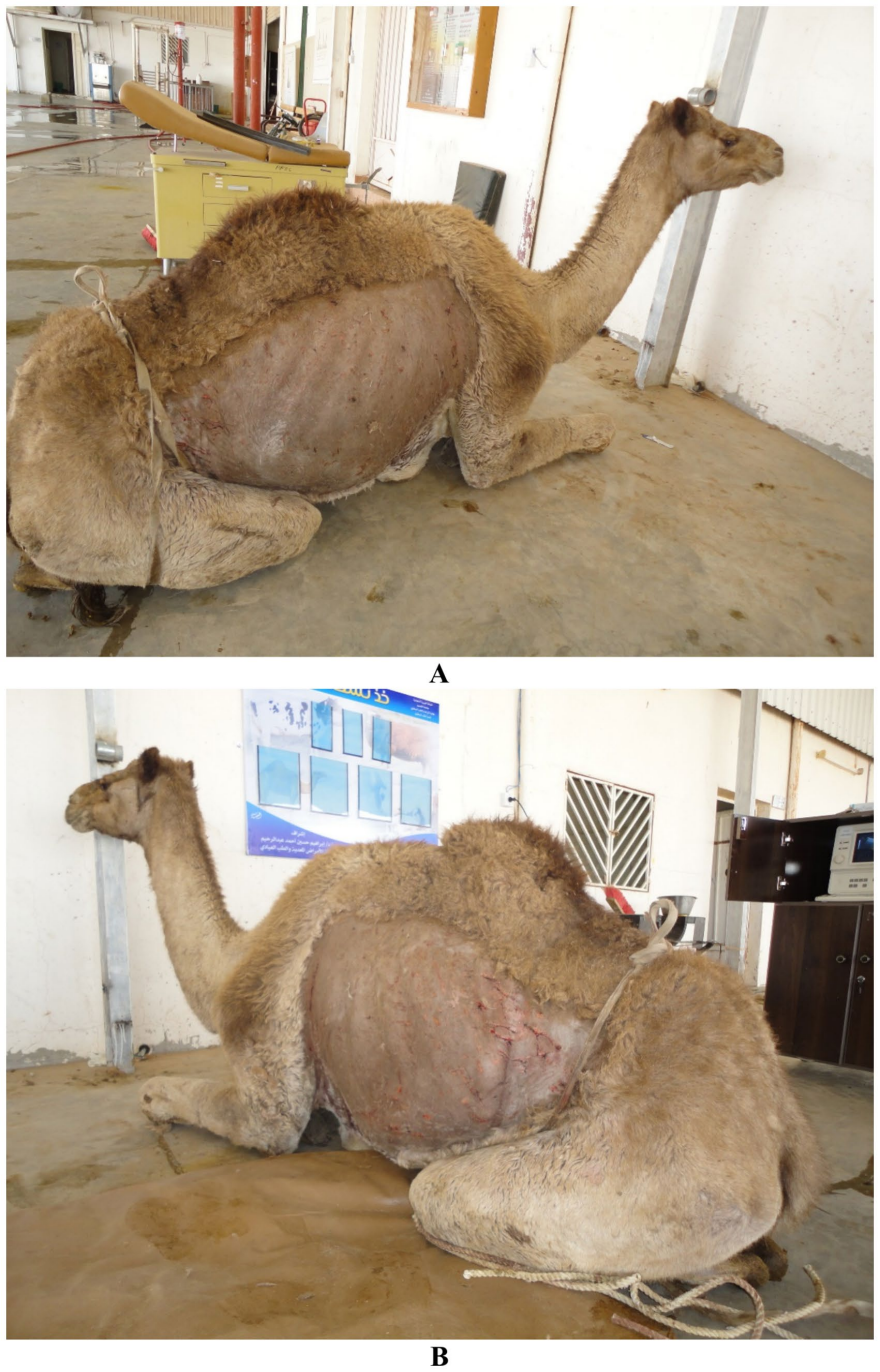
Preparation for cardiopulmonary ultrasonographic examination in a camel. A female dromedary is shown in sternal recumbency with clipped and shaved thoracic regions on both the right **(A)** and left **(B)** sides, enabling optimal contact for transducer placement. Proper preparation enhances image quality during echocardiographic and thoracic assessments [modified from Tharwat (32)].

Given the size and temperament of camels, most thoracic and cardiac ultrasound examinations are performed with the animal in a sitting position, as this posture allows easier access to the lateral thoracic wall and better visualization of gravity-dependent structures such as the pleural space (51). Manual restraint using halters and lead ropes is generally sufficient for calm or habituated animals, particularly when conducted in familiar environments. However, in more excitable or uncooperative individuals, the use of a crush or chute system may be necessary to minimize movement without inducing stress-related artifacts in cardiovascular parameters (18). Sedation with agents such as xylazine may be employed cautiously in clinical settings, although its depressant effects on cardiac function and respiratory drive must be considered when evaluating cardiopulmonary parameters (44). For focused cardiac assessments, access to the right and left thoracic windows may require repositioning the forelimbs slightly forward to expose intercostal spaces, especially in well-muscled or obese animals (49). Adequate surface preparation, including clipping and application of coupling gel, remains essential regardless of posture (Figure 3). Ultimately, selecting the optimal combination of restraint technique and animal positioning depends on the clinical context, the region being examined, and the behavioral characteristics of the individual camel, all of which influence the feasibility and diagnostic yield of thoracic ultrasonography (11).

### 3.5 Scanning windows and approaches

The selection of appropriate scanning windows and systematic approaches is fundamental to obtaining high-quality ultrasonographic images of the camel thorax. In dromedary camels, thoracic ultrasound is typically performed through the intercostal spaces along both lateral thoracic walls, with the animal in a sitting position to allow natural displacement of intrathoracic organs (8). For pulmonary assessment, the dorsal, middle, and ventral thoracic zones are explored bilaterally from the 3rd to the 11th intercostal spaces, using longitudinal and transverse probe orientations to evaluate pleural surfaces, lung fields, and diaphragm contours (7). Specific landmarks such as the scapula, elbow, and costochondral junction help guide transducer placement and maintain consistency across examinations. Cardiac imaging in camels is primarily achieved through right and left parasternal windows. The right parasternal long-axis and short-axis views provide access to the four cardiac chambers, interventricular septum, and great vessels, while left-sided views can offer additional perspectives when acoustic windows are favorable (31). These approaches are adapted from techniques used in equine and bovine cardiology but require modification due to the camel’s deeper chest and heavier musculature (32). The use of phased-array or microconvex probes facilitates image acquisition in narrow intercostal spaces, particularly for dynamic structures such as cardiac valves. A methodical approach to scanning, combined with clear knowledge of regional anatomy, enhances diagnostic accuracy and supports the expanding role of ultrasonography in camel cardiopulmonary medicine (32).

### 3.6 Limitations

While ultrasonography offers significant advantages in the assessment of thoracic and cardiac structures in camels, it is not without limitations. One of the primary challenges is the presence of air within the lungs, which reflects ultrasound waves and prevents visualization of normal pulmonary parenchyma. This restricts sonographic evaluation to pleural surfaces and peripheral lung zones unless pathological changes, such as consolidation or effusion, create acoustic windows for deeper imaging (47). Additionally, the camel’s thick body wall, dense musculature, and coarse hair can reduce image quality, particularly when using higher-frequency transducers with limited tissue penetration (11). Common artifacts encountered during scanning include reverberation (A-lines), mirror image effects, comet-tail artifacts, and acoustic shadowing from ribs—each of which must be correctly interpreted to avoid diagnostic errors (45). Motion artifacts from respiration and cardiac pulsation, as well as animal movement due to inadequate restraint, can further compromise image acquisition and interpretation. Moreover, the absence of species-specific reference values and standardized scanning protocols in camels can limit diagnostic consistency across different operators and institutions (32). Inter-operator variability, especially in field settings where environmental conditions and operator experience vary widely, can further impact the reproducibility and accuracy of ultrasonographic assessments. Consistent training and standardized protocols are therefore essential to minimize such variability and improve diagnostic reliability (32). Despite these constraints, understanding the nature and origin of sonographic artifacts and adapting imaging techniques accordingly can significantly enhance the reliability of ultrasonography as a diagnostic tool in camel cardiopulmonary evaluation.

## 4 Normal ultrasonographic findings in healthy dromedary camels

### 4.1 Normal cardiac ultrasonographic anatomy

In healthy dromedary camels, cardiac ultrasonography reveals species-specific anatomical features. However, a thorough understanding of these is essential to accurately distinguish normal findings from pathological conditions (34). The heart is best visualized from the right and left parasternal windows, with the right side generally offering more consistent imaging due to reduced interference from the forelimb musculature and lung overlap (11, 14). Using a phased-array or microconvex transducer with a frequency of 2.5–5 MHz, the right parasternal long-axis view typically displays the left and right ventricles, left atrium, right atrium, interventricular septum, and mitral and tricuspid valves in dynamic motion (50). In the short-axis view, cross-sectional images of the ventricles and great vessels—such as the aorta and pulmonary artery—can be appreciated, allowing for assessment of wall thickness and chamber dimensions. M-mode imaging provides quantitative parameters including left ventricular internal diameter, interventricular septal thickness, and fractional shortening, which are crucial for evaluating systolic function in clinical settings (11). Normal echocardiographic findings in camels include a relatively thick myocardial wall, slow heart rate (typically 35–45 bpm at rest), and uniform endocardial motion without regurgitation or turbulence on Doppler assessment (43, 44). The cardiac apex is directed caudally and slightly to the left, similar to large ruminants, but the heart lies more vertically within the thoracic cavity. These anatomical and functional norms provide the essential baseline against which pathological deviations can be interpreted in camel cardiology (11).

#### 4.1.1 Right parasternal ultrasonograms

**Figure 4:**
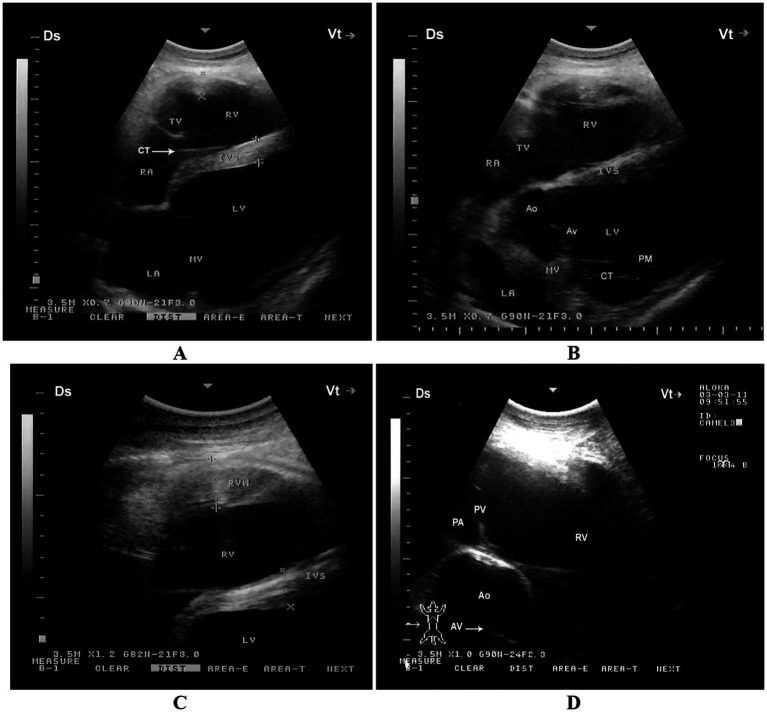
Right parasternal echocardiographic views in a healthy dromedary camel. **(A)** Caudal long-axis four-chamber view displaying the left and right ventricles with visible tricuspid chordae tendineae (arrow). This view is valuable for assessing chamber size, tricuspid valve morphology, and right ventricular function. **(B)** Caudal long-axis view including the left ventricular outflow tract, allowing evaluation of aortic valve function and potential outflow obstructions. **(C)** Short-axis view at the level of the ventricles showing both ventricles in cross-section, useful for assessing ventricular wall motion and relative chamber dimensions. **(D)** Cranial long-axis view at the third intercostal space highlighting the right ventricular outflow tract and pulmonary artery, which aids in evaluating pulmonary valve structure and detecting pulmonary hypertension. Ds, dorsal; Vt, ventral; RV, right ventricle; PA, pulmonary artery; PV, pulmonary valve; Ao, aorta; AV, aortic valve; RVW, right ventricular wall; LV, left ventricle; IVS, interventricular septum; LA, left atrium; RA, right atrium; TV, tricuspid valve; MV, mitral valve; CT, chordae tendineae; PM, papillary muscles [adapted from Tharwat et al. (11)].

When the probe is positioned longitudinally in the 5th intercostal space (ICS) with a slight clockwise rotation, or placed perpendicularly in the 4th ICS, a caudal long-axis four-chamber view can be obtained. This view includes the right and left ventricles, right and left atria, the interventricular septum, as well as the tricuspid and mitral valves. In some camels, this same position may yield a hybrid view combining features of the four-chamber and left ventricular outflow tract (LVOT) views. A slight clockwise rotation of the probe in the 4th ICS can provide a short-axis view of the cardiac ventricles. Additionally, placing the transducer in the 3rd ICS enables visualization of the right ventricular outflow tract (RVOT), allowing imaging of the right ventricle, pulmonary valve, pulmonary artery, aorta, and aortic valve (11) (Figure 4).

#### 4.1.2 Left parasternal ultrasonograms

**Figure 5:**
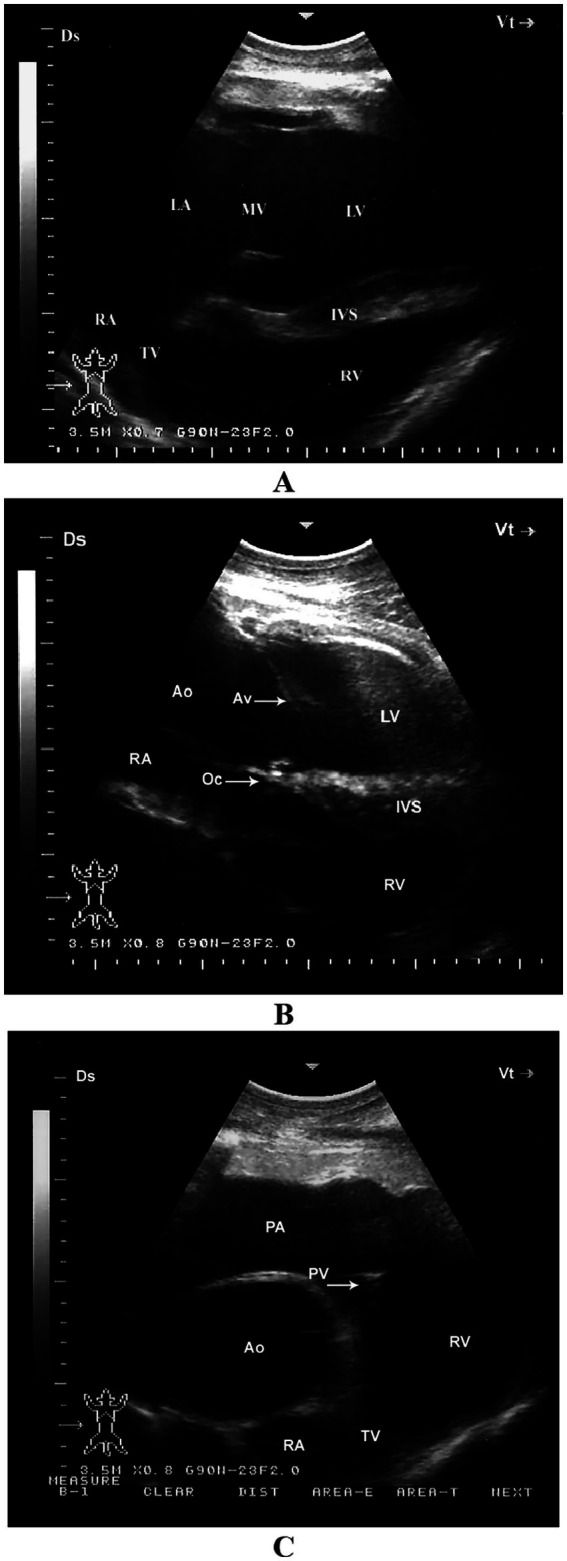
Left parasternal echocardiographic views in a healthy dromedary camel. **(A)** Caudal long-axis view displaying all four cardiac chambers and atrioventricular valves, enabling comprehensive assessment of chamber dimensions and valve function. **(B)** Left ventricular outflow tract view with the aortic valve seen as a thin echoic line in cross-section; this view assists in evaluating aortic valve morphology and detecting stenosis or regurgitation. **(C)** Cranial long-axis view highlighting the right ventricular outflow tract and aortic root, useful in identifying structural abnormalities in the outflow tract and assessing great vessel alignment. Ds, dorsal; Vt, ventral; RV, right ventricle; RA, right atrium; TV, tricuspid valve; Ao, aorta; PA, pulmonary artery; PV, pulmonary valve; LV, left ventricle; IVS, interventricular septum; AV, aortic valve; Oc, ossa cordis; LA, left atrium; MV, mitral valve [adapted from Tharwat et al. (11)].

When the probe is placed longitudinally in the 4th or 5th intercostal space (ICS) and angled slightly caudodorsally, it allows visualization of the ventricles, atria, and atrioventricular valves. The LVOT can be imaged from the 4th ICS by directing the probe slightly more cranially and rotating it slightly counterclockwise. The RVOT is best visualized from the 3rd ICS. In this position, the right ventricle, tricuspid valve, right atrium, an oblique section of the aorta, and the pulmonary artery can be clearly seen (11) (Figure 5).

### 4.2 Chamber size, wall thickness, valves

Echocardiographic evaluation of cardiac morphology in healthy dromedary camels reveals specific baseline parameters related to chamber dimensions, myocardial wall thickness, and valvular motion, all of which are critical for identifying structural or functional abnormalities. In adult camels, the left ventricular internal diameter during diastole typically ranges between 5.0 to 6.5 cm, while the interventricular septal thickness and left ventricular free wall measure approximately 1.2 to 1.8 cm, values that reflect the camel’s adaptation to sustained circulatory demands in arid environments (11, 50). Right ventricular dimensions are smaller and more variable, often depending on hydration status and physiological workload. The cardiac chambers show symmetric geometry, with preserved systolic function evidenced by normal fractional shortening values of 28–40%, as assessed via M-mode imaging (14, 32). Valvular structures, including the mitral, tricuspid, aortic, and pulmonary valves, are typically thin, pliable, and free of vegetative or calcific changes in healthy individuals. Color and spectral Doppler imaging in normal camels reveal smooth, laminar flow across all valves with no significant regurgitation or stenotic jets (43, 44). Of note, the camel mitral and tricuspid annuli are relatively large and demonstrate mild physiological regurgitation in some cases, especially during periods of stress or restraint, which should not be misinterpreted as pathological (11). Establishing these baseline echocardiographic indices is essential for the differentiation of normal variants from early pathological changes in clinical cardiology.

### 4.3 Normal pulmonary ultrasound findings

In healthy dromedary camels, thoracic ultrasonography provides valuable insights into the superficial pulmonary structures, despite the inherent limitations of ultrasound in evaluating aerated lung parenchyma. Under normal conditions, the lung surface appears as a hyperechoic, smoothly curving pleural line located just beneath the ribs, which moves synchronously with respiration—a phenomenon referred to as the “lung sliding” sign (7, 14). This dynamic motion, combined with the presence of reverberation artifacts known as A-lines, indicates the presence of normally aerated lung tissue and the absence of significant pleural or parenchymal disease (47). In the absence of pathology, the interstitial space between the parietal and visceral pleura is indistinct, with no detectable fluid accumulation or thickening. Lung comets or B-lines, which are vertical hyperechoic artifacts arising from the pleural line, may be seen in small numbers and are considered physiological when limited in distribution (31). Importantly, due to the camel’s narrow intercostal spaces and thick thoracic wall, optimal imaging of the lung surface is often restricted to the dorsal and mid-thoracic zones from the 4th to 11th intercostal spaces. The diaphragm appears as a curvilinear echogenic structure caudal to the lung field and is visualized best in the caudoventral thorax. Normal findings also include the absence of pleural effusion, pulmonary masses, or consolidation, which would otherwise disrupt the regular artifact pattern and pleural sliding (32).

**Figure 6:**
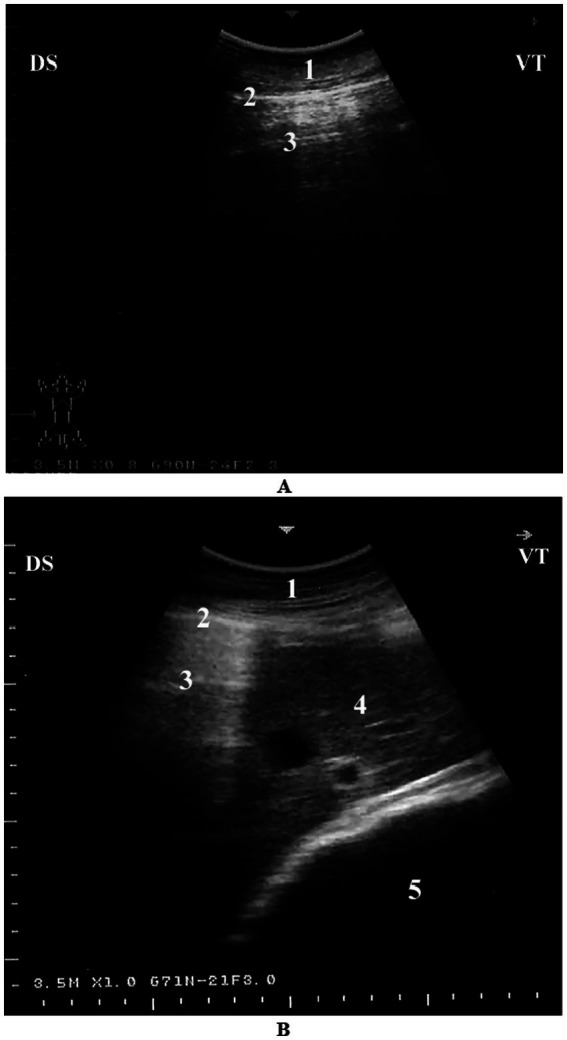
Normal lung ultrasonography in a camel. **(A)** Intercostal view from the right 8th intercostal space showing a typical reverberation pattern beneath the pleural line. **(B)** Cross-sectional image from the right 6th intercostal space showing normal lung (with reverberation), liver, and omasum. 1 – Thoracic wall; 2 – Pleura; 3 – Reverberation artifacts; 4 – Liver; 5 – Omasum; Ds – Dorsal; Vt – Ventral [modified from Tharwat (7)].

Sonographically, the pulmonary surface is visible from the 4th to the 11th intercostal spaces. The dorsal border of the lung maintains a relatively consistent distance from the dorsal midline. However, between the 4th and 7th intercostal spaces, the dorsal portion of the lung is obscured by the scapula, increasing the distance to the dorsal midline in these regions. The ventral lung border follows a caudodorsal trajectory, resulting in a progressive decrease in the distance between the dorsal midline and the ventral lung border from cranial to caudal. This border is most extensive at the 4th ICS and narrowest at the 11th ICS. The dorsoventral dimension of the lung is greatest at the 8th ICS and gradually diminishes both caudally beyond the 11th ICS and cranially toward the 4th ICS (Table 1). It is important to note that the measured lung size in these intercostal spaces reflects only the portion accessible to ultrasonographic examination and does not represent the actual lung dimensions. The echogenic line visible on the lung surface, formed by the costal and parietal pleurae, measures approximately 1 to 4 mm in thickness (7) (Figure 6). Recognizing these normal ultrasonographic features is essential to avoid false-positive interpretations and to serve as a comparative baseline for detecting pulmonary pathology in clinical and field settings.

**Table 1:** Internal echocardiographic measurements in healthy adult camels (*n* = 22)*.

Variable	Min	Max	Mean	SD	CV
RVd (cm)	4.3	8.0	5.3	1.2	22%
RVs (cm)	4.2	4.6	4.1	0.4	10%
LVd (cm)	9.9	15.5	11.8	1.6	14%
LVs (cm)	7.8	9.4	8.2	0.6	10%
RAd (cm)	5.0	6.5	5.9	0.5	10%
RAs (cm)	3.0	4.5	3.9	0.4	10%
LAd (cm)	6.8	8.8	7.6	0.6	10%
LAs (cm)	4.7	6.4	5.6	0.5	10%
RVWd (cm)	1.5	2.4	1.8	0.2	10%
RVWs (cm)	1.2	2.0	1.5	0.2	13%
IVSd (cm)	1.7	3.0	2.1	0.3	14%
IVSs (cm)	1.2	3.9	2.8	0.7	30%
LVWd (cm)	1.8	3.4	2.8	0.4	14%
LVWs (cm)	1.1	2.4	1.9	0.4	21%
AOd (cm)	6.0	8.2	7.0	0.8	11%
PAd (cm)	6.0	9.7	8.1	1.2	15%
TVDs (cm)	2.6	6.8	4.1	1.1	27%
MVDs (cm)	4.3	7.8	6.2	1.0	16%

N, number of the camels; Min, minimum value; Max, maximum value; SD, standard deviation; CV, coefficient of variation; RV, right ventricular diameter; LV, left ventricular diameter; RA right atrium diameter; LA, left atrium diameter; RVW, right ventricular wall thickness; IVS, interventricular septal thickness; LVW, left ventricular wall thickness; AO, aortic diameter; PA, pulmonary artery diameter; TVD, tricuspid valve diameter; MVD, mitral valve diameter; d, diastole; s, systole. *Tharwat et al. (11).

### 4.4 Pleural line, lung sliding, absence of pathology and reference values for echocardiographic measurements

The pleural line is one of the most critical ultrasonographic landmarks in the assessment of the camel thorax, serving as a key indicator of pulmonary integrity. In healthy dromedary camels, the pleural line appears as a bright, hyperechoic horizontal band situated just beneath the rib shadows and represents the interface between the parietal and visceral pleurae (7). This line moves rhythmically with respiration in a phenomenon termed “lung sliding,” which reflects normal visceral-parietal pleural apposition and confirms the absence of pneumothorax or pleural adhesions (47). The presence of smooth, uninterrupted lung sliding is considered a hallmark of healthy lung function and should be consistently visible in all accessible intercostal spaces under physiological conditions. In addition, the absence of subpleural irregularities, fluid lines, or discontinuities in the pleural contour suggests that the pleural surfaces are free of effusion, fibrin, or mass effect (31). A-line artifacts—repeating horizontal reverberations beneath the pleural line—are another normal finding and indicate underlying air-filled alveoli, while the absence of significant B-lines reinforces the diagnosis of normally aerated lungs (14).

Proper identification of these sonographic features is particularly important in camels, where external signs of thoracic disease may be subtle and masked by stoic behavior. As such, recognition of a well-defined pleural line with regular lung sliding and typical artifact profiles forms the foundation for distinguishing normal from pathological thoracic ultrasound findings in clinical and herd-health evaluations (8). Establishing species-specific reference values for echocardiographic measurements is essential for accurate interpretation of cardiac function in dromedary camels, given their unique cardiothoracic anatomy and physiological adaptations. The minimum, maximum, and mean values, along with standard deviations and coefficients of variation for internal echocardiographic measurements in healthy adult dromedary camels, are summarized in Table 1 (11).

## 5 Ultrasonography in the diagnosis of cardiopulmonary diseases

### 5.1 Pulmonary and pleural diseases

#### 5.1.1 Pneumonia

**Figure 7:**
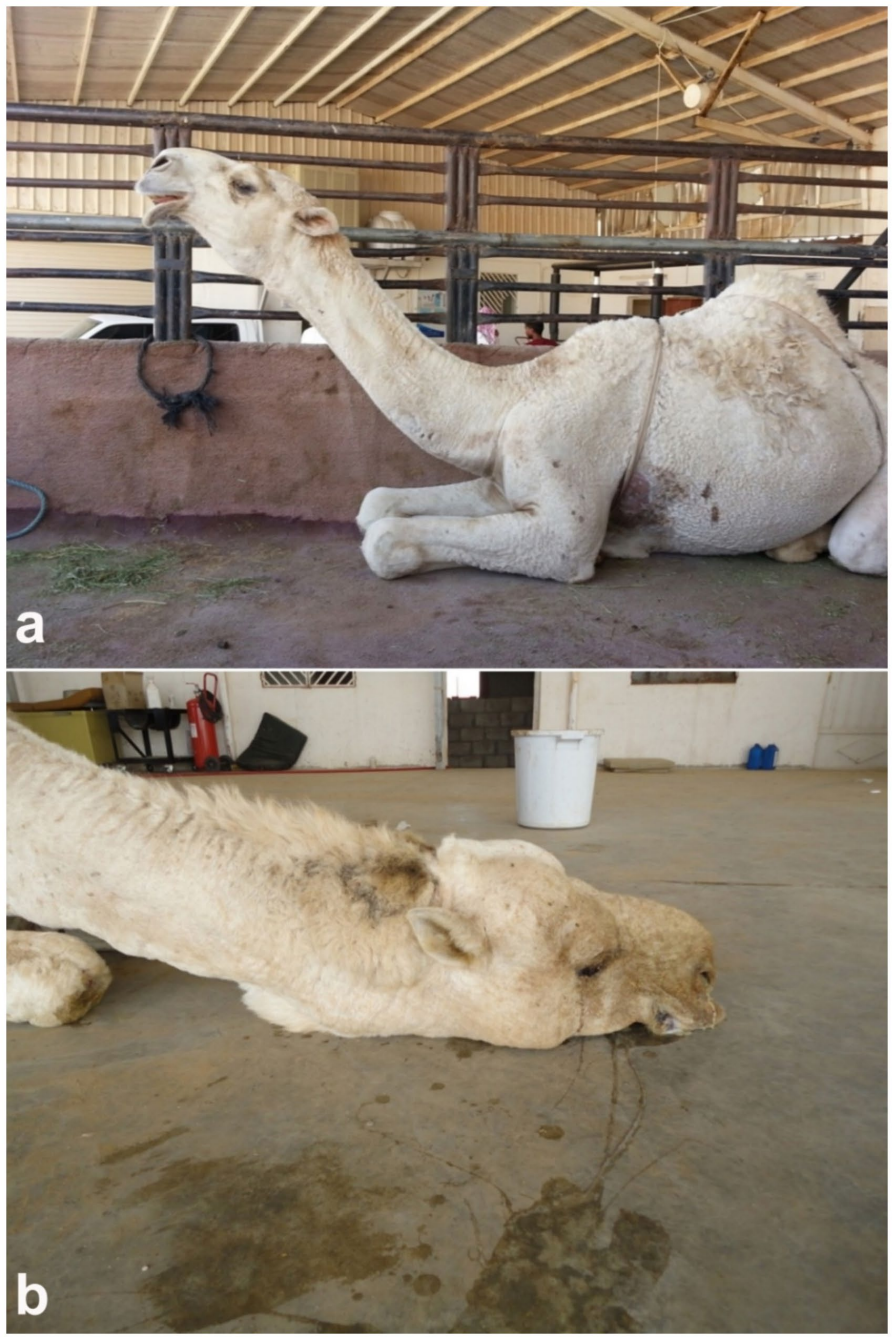
Clinical signs of pneumonia in dromedary camels. **(a)** Female camel displaying extended head and neck posture, characteristic of respiratory distress. **(b)** Male camel in a recumbent position with head and neck resting on the ground, indicating advanced pulmonary compromise [modified from Tharwat and Al-Sobayil (20)].

Pneumonia remains one of the most prevalent and economically significant respiratory disorders in dromedary camels, often presenting with nonspecific clinical signs that may delay early detection and treatment. Typical signs include reduced appetite, lethargy, pyrexia, nasal discharge, coughing, tachypnea, and increased respiratory effort, which may progress to respiratory distress in advanced stages (52–54). In camels, as in other domesticated animals, the etiology of pneumonia can be infectious—caused by bacteria, viruses, fungi, or parasites—or non-infectious, such as aspiration of foreign material or exposure to toxins through hematogenous spread or inhalation. The majority of infectious pneumonias are attributed to opportunistic bacterial pathogens (55). Despite their physiological resilience and adaptation to arid, challenging environments, camels remain susceptible to respiratory disorders, which can result in significant economic losses. These include reduced productivity, increased veterinary expenditures, carcass condemnation, and mortality (56–58). In severe or terminal stages, affected animals may assume a posture with the head and neck fully extended on the ground, and exhibit open-mouth breathing with flared nostrils (20) (Figure 7).

**Figure 8:**
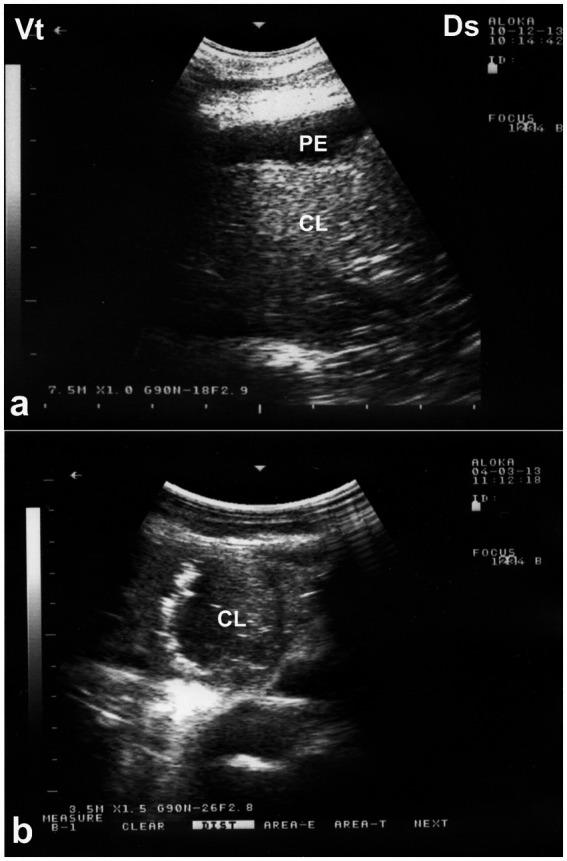
Ultrasonographic appearance of lung consolidation in pneumonic dromedary camel calves. **(a)** Longitudinal ultrasonographic image showing hypoechoic, consolidated lung tissue adjacent to the thoracic wall, consistent with lobar pneumonia. Anechoic pleural effusion (PE) is visible between the visceral and parietal pleura, suggesting an exudative process. **(b)** High-resolution image demonstrating multiple small hyperechoic foci scattered within the consolidated parenchyma, representing trapped air or gas. These foci cause distal acoustic shadowing, indicative of bronchograms or air-filled bronchioles within non-aerated lung. Ds – Dorsal; Vt – Ventral [modified from Tharwat and Al-Sobayil (8)].

**Figure 9:**
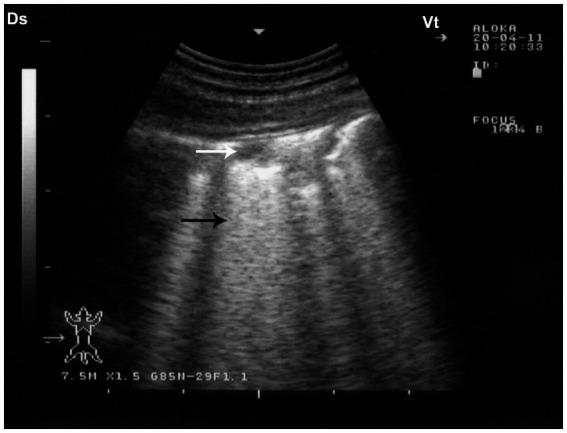
Sonographic findings in drenching pneumonia. The lung surface exhibits multiple small hypoechoic zones (white arrow) interpreted as superficial fluid alveolograms, along with characteristic comet-tail artifacts (black arrow), indicative of interstitial-alveolar pathology. Ds – Dorsal; Vt – Ventral [modified from Tharwat and Al-Sobayil (8)].

Given the limited sensitivity of auscultation in detecting deep pulmonary lesions in large animals, ultrasonography has emerged as a valuable diagnostic tool in the evaluation of camelid thoracic pathology (8, 14). Thoracic ultrasonography enables real-time, non-invasive visualization of superficial pulmonary and pleural abnormalities, facilitating the early diagnosis and monitoring of pneumonia. In affected camels, sonographic features often include pulmonary consolidation with hepatization, characterized by a homogenous echotexture resembling liver tissue, and occasional detection of fluid bronchograms or pleural effusions (8). In cases of aspiration or drenching pneumonia, comet-tail artifacts and superficial hypoechoic zones—termed superficial fluid alveolograms—may be evident along the pleural surface (8). Ultrasonography has proven particularly beneficial in differentiating between infectious and non-infectious etiologies, assessing the extent of pulmonary involvement, and guiding clinical decisions, especially when radiography is impractical due to the camel’s size and field conditions (14). Ultrasonographic examination of the affected lungs typically reveals areas of pulmonary consolidation, occasionally accompanied by pleural effusion. The affected, non-aerated lung tissue often exhibits an echotexture resembling that of hepatic parenchyma (8) (Figure 8). Occasionally, fluid bronchograms and anechoic vascular structures can be visualized within the hepatized regions of the lung. The aerated lung tissue located beneath the consolidated areas is typically identified by the presence of faint, ill-defined, and blurred reverberation artifacts. In cases of drenching pneumonia, ultrasonographic assessment reveals hypoechoic regions on the pulmonary surface, indicative of “superficial fluid alveolograms,” often accompanied by comet-tail artifacts (8) (Figure 9).

#### 5.1.2 Pulmonary abscesses

Pulmonary abscesses, although less frequently reported than pneumonia, represent a significant manifestation of chronic respiratory infection in dromedary camels, often arising as sequelae to unresolved bronchopneumonia or aspiration events. Clinically, affected camels may exhibit prolonged illness marked by intermittent fever, progressive weight loss, chronic coughing, labored breathing, and bilateral nasal discharge, which may be fetid or purulent in nature (54). These signs are often subtle and nonspecific in the early stages, contributing to diagnostic delays and disease progression (18).

**Figure 10:**
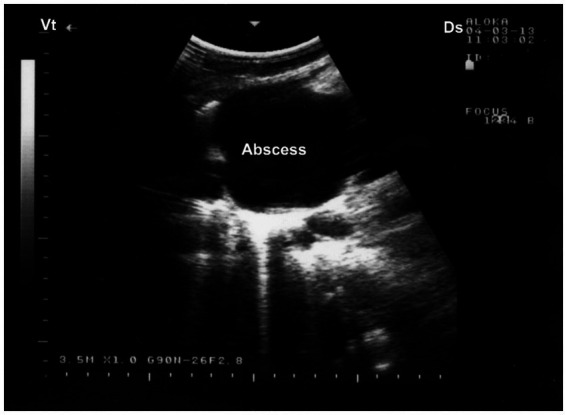
Ultrasonographic visualization of a lung abscess. A subpleural, well-demarcated hypoechoic lesion is noted immediately beneath the chest wall, bordered by a hyperechoic capsule. Acoustic enhancement (AE) is visible distal to the lesion, suggesting fluid-filled content consistent with abscessation. Ds – Dorsal; Vt – Ventral [modified from Tharwat (32)].

Ultrasonography serves as a valuable diagnostic adjunct in the detection and characterization of pulmonary abscesses, particularly in field settings where radiography is not feasible. Sonographic features typically include well-demarcated hypoechoic to anechoic cavities within consolidated lung tissue, frequently containing internal echogenic material suggestive of purulent content, cellular debris, or necrotic tissue (59). In some cases, gas echoes or reverberation artifacts may be observed within the abscess cavity, indicative of anaerobic infection. The surrounding lung parenchyma often displays hepatization or irregular pleural surfaces, and pleural thickening or adhesions may also be noted (20). In chronic cases, capsulated abscesses may exhibit peripheral vascularization detectable with Doppler ultrasonography, aiding in differentiation from neoplastic or granulomatous lesions. As such, ultrasonography not only facilitates early and non-invasive diagnosis but also provides essential guidance for prognosis, therapeutic planning, and, when applicable, ultrasound-guided drainage or sampling (8). Ultrasonographic examination of the affected lung typically reveals a hyperechogenic pleural surface, pulmonary parenchyma of medium echogenicity, and a heterogeneous pattern resembling liver tissue. Within the compressed lung parenchyma, relatively well-defined abscesses appear as round to ovoid anechoic regions. The abscess capsule is seen as a thin, reflective band, with acoustic enhancement visible beneath the lesion (32) (Figure 10).

#### 5.1.3 Pneumothorax and emphysema

Pneumothorax, defined as the accumulation of free air within the pleural cavity, represents a critical and potentially life-threatening condition in dromedary camels, often resulting from thoracic trauma, penetrating injuries, rib fractures, or as a complication of pulmonary pathology such as abscess rupture or severe pneumonia (52). Clinically, camels with pneumothorax may present with acute respiratory distress, characterized by rapid and shallow breathing, open-mouth respiration, cyanosis, and reduced or absent breath sounds on auscultation over the affected hemithorax. Affected animals may adopt a characteristic stance with the neck extended and nostrils flared, while in severe bilateral cases, signs of hypoxia and recumbency may develop rapidly (53).

Hypersensitivity or allergic respiratory disease syndrome results in the enlargement of air spaces distal to the terminal bronchioles, accompanied by the destruction of alveolar walls without associated fibrosis. Common etiologies include chronic obstructive pulmonary disease, acute interstitial pneumonia, large pulmonary abscesses, strenuous exercise, and idiopathic causes. Pulmonary emphysema is associated with pulmonary edema, congestion, interstitial emphysema, and alterations in alveolar structure. Clinically, it is characterized by marked respiratory distress, frothy salivation, and open-mouth breathing (18). Diagnosis is based on physical examination findings such as hyperresonance on percussion, enlargement of the lung field, and diminished vesicular breath sounds (58).

**Figure 11:**
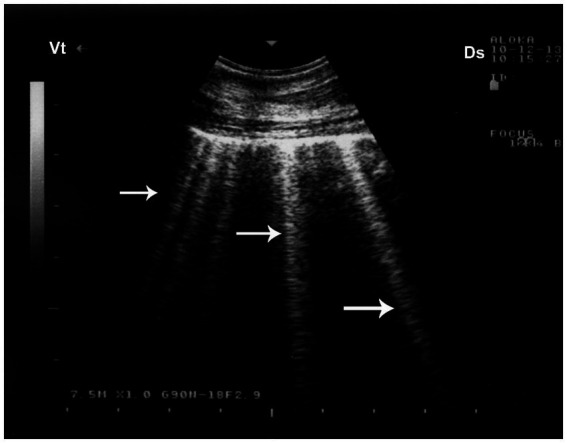
Pulmonary emphysema detected by ultrasonography. Numerous bright echogenic reverberation bands (white arrows) arising from the pleural surface represent comet-tail artifacts, commonly seen in emphysematous lung changes. Ds – Dorsal; Vt – Ventral [modified from Tharwat (32)].

Ultrasonography plays a pivotal role in the prompt diagnosis of pneumothorax, particularly in field settings where conventional imaging is limited. The absence of normal lung sliding—a shimmering motion at the pleural line due to apposition of visceral and parietal pleura—is a hallmark sonographic sign of pneumothorax (8). In camels, thoracic ultrasonography is especially valuable due to their large thoracic volume and the difficulty of obtaining clear radiographic images in adult animals. Early detection through ultrasonography facilitates timely intervention, such as thoracocentesis or surgical management, significantly improving clinical outcomes. Ultrasonographic evaluation in camels with pulmonary emphysema reveals numerous comet-tail artifacts—bright, closely spaced echo bands originating at the lung surface and extending perpendicularly into the lung parenchyma (32) (Figure 11).

#### 5.1.4 Pleural effusion

Pleural effusion, the pathological accumulation of fluid within the pleural cavity, is an uncommon but clinically significant condition in dromedary camels. It may arise secondary to bacterial or parasitic infections, neoplastic processes, thoracic trauma, or extension of pulmonary disease such as pneumonia or abscessation (52). Clinically, camels with pleural effusion often present with nonspecific but progressive signs, including reduced appetite, weight loss, labored breathing, and reluctance to move. On auscultation, muffled heart and lung sounds may be appreciated over ventral thoracic regions, and in advanced cases, affected animals may exhibit orthopnea, extended neck posture, and audible respiratory effort (20). The condition is typically secondary to pleuritis, pericarditis, or right-sided heart failure. Clinical signs include inspiratory dyspnea, absence of respiratory sounds in the lower thoracic region, and a dull percussion sound in the same area—findings that are considered diagnostic (18).

**Figure 12:**
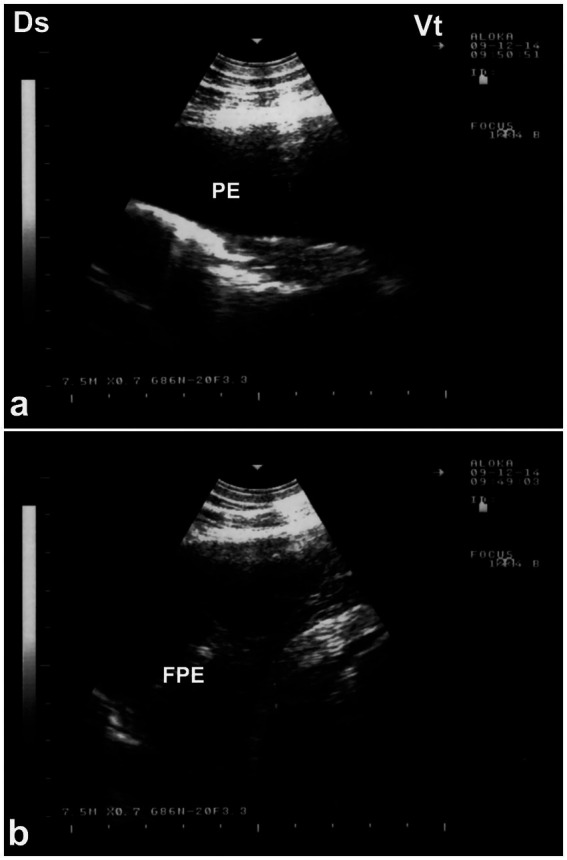
Ultrasonographic evidence of pleural effusion in a dromedary camel calf. **(a)** Longitudinal ultrasonographic image of the right hemithorax showing an anechoic pleural space between the collapsed lung surface and the thoracic wall, consistent with a free, transudative pleural effusion (PE). The underlying lung is partially visualized as a compressed, hypoechoic structure. **(b)** Left thoracic ultrasound image demonstrating fibrinous pleural effusion (FPE), characterized by multiple echogenic fibrin strands floating within the anechoic fluid. These suspended echogenic bands are indicative of an exudative or inflammatory process, typically associated with infectious pleuropneumonia. Ds – Dorsal; Vt – Ventral [modified from Tharwat (32)].

Ultrasonography is considered the most reliable and practical tool for diagnosing pleural effusion in camels, particularly in the absence of accessible radiographic facilities. Sonographic findings typically include an anechoic or hypoechoic fluid accumulation between the visceral and parietal pleura, with displacement or compression of adjacent lung tissue. Fibrin strands, septations, and floating echogenic debris may also be visualized, particularly in exudative or chronic effusions (8). Ultrasonography not only enables accurate assessment of the volume and nature of pleural fluid but also guides safe and effective thoracocentesis, fluid sampling, or drainage procedures. Furthermore, it facilitates serial monitoring of disease progression and response to therapy, thereby enhancing clinical decision-making and prognosis (32). In dromedaries presenting with clinical signs of pleural effusion, ultrasonographic examination reveals hypoechoic to anechoic fluid within the pleural cavity, located between the parietal pleura, diaphragm, and lung. Echogenic strands or fibrin may be visible within this fluid. The accumulation of pleural fluid causes compression atelectasis, particularly in the cranial lung lobes, which appear hypoechoic. Entrapped air in larger bronchi appears hyperechoic and is often accompanied by comet-tail artifacts. Fibrin is visualized as thin, filamentous strands floating within the effusion, loosely attached to the pleural surfaces. It is also common to observe fluid pockets separated by fibrin strands (32) (Figure 12).

#### 5.1.5 Pleurisy and pleuropneumonia

Pleurisy, or pleuritis, refers to inflammation of the pleural membranes and is often associated with or progresses to pleuropneumonia when concurrent pulmonary parenchymal infection is present. Inspiratory dyspnea is the most prominent clinical sign. Diagnostic indicators include the absence of respiratory sounds in the lower thoracic region and dullness on percussion in the same area. A definitive diagnosis relies on radiographic imaging and thoracocentesis (19). Determining the nature of the pleural fluid is essential, as it may be a modified transudate or an exudate. Treatment depends on the underlying cause. While thoracocentesis can be used to remove excess fluid, the primary therapeutic goal is to prevent recurrence. The prognosis is poor in cases involving neoplasia. Infectious pleuritis should be managed with broad-spectrum antibiotics until culture and sensitivity results are available (18). In dromedary camels, pleurisy and pleuropneumonia are typically secondary to bacterial infections, parasitic infestations, or complications from untreated pneumonia and thoracic trauma (19). Common clinical signs include fever, anorexia, shallow or painful respiration, and a guarded posture. Audible pleural friction rubs may be present early, while advanced stages often show diminished breath sounds and signs of respiratory distress, including nasal flaring and extended neck posture (60).

**Figure 13:**
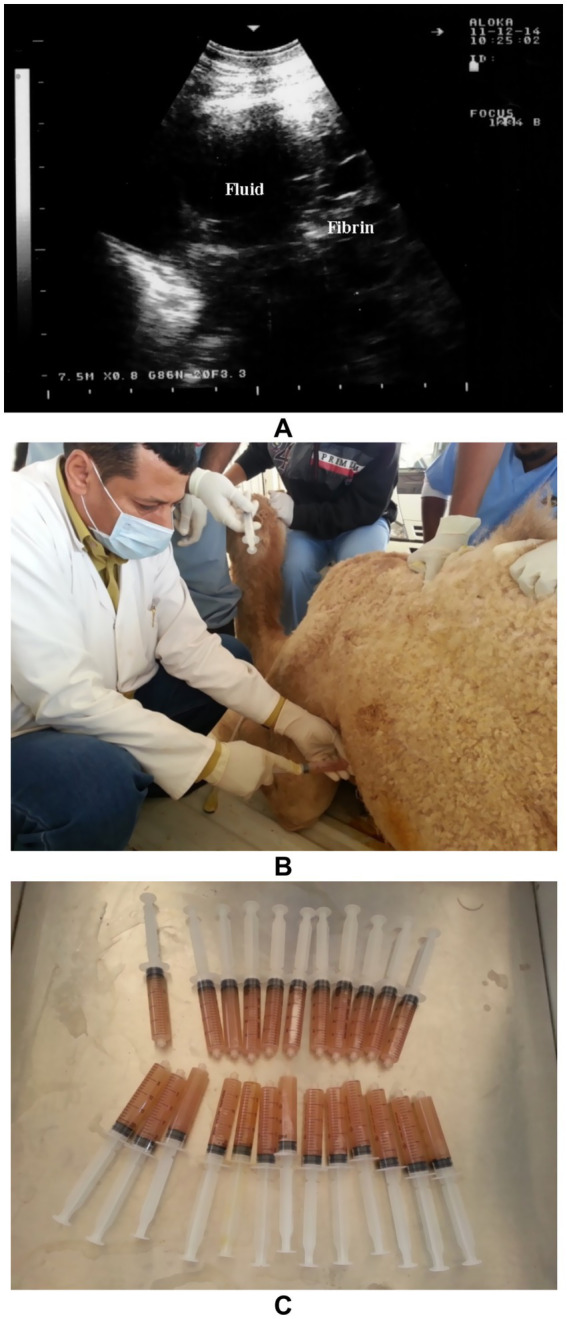
Thoracic ultrasonography in a female camel diagnosed with pleuropneumonia showing anechoic pleural fluid containing fibrin strands **(A)**. Thoracocentesis being performed on the left thoracic cavity **(B)**, and collection of pleural exudates for storage and further analysis **(C)** [modified from Tharwat and Al-Sobayil (8)].

**Figure 14:**
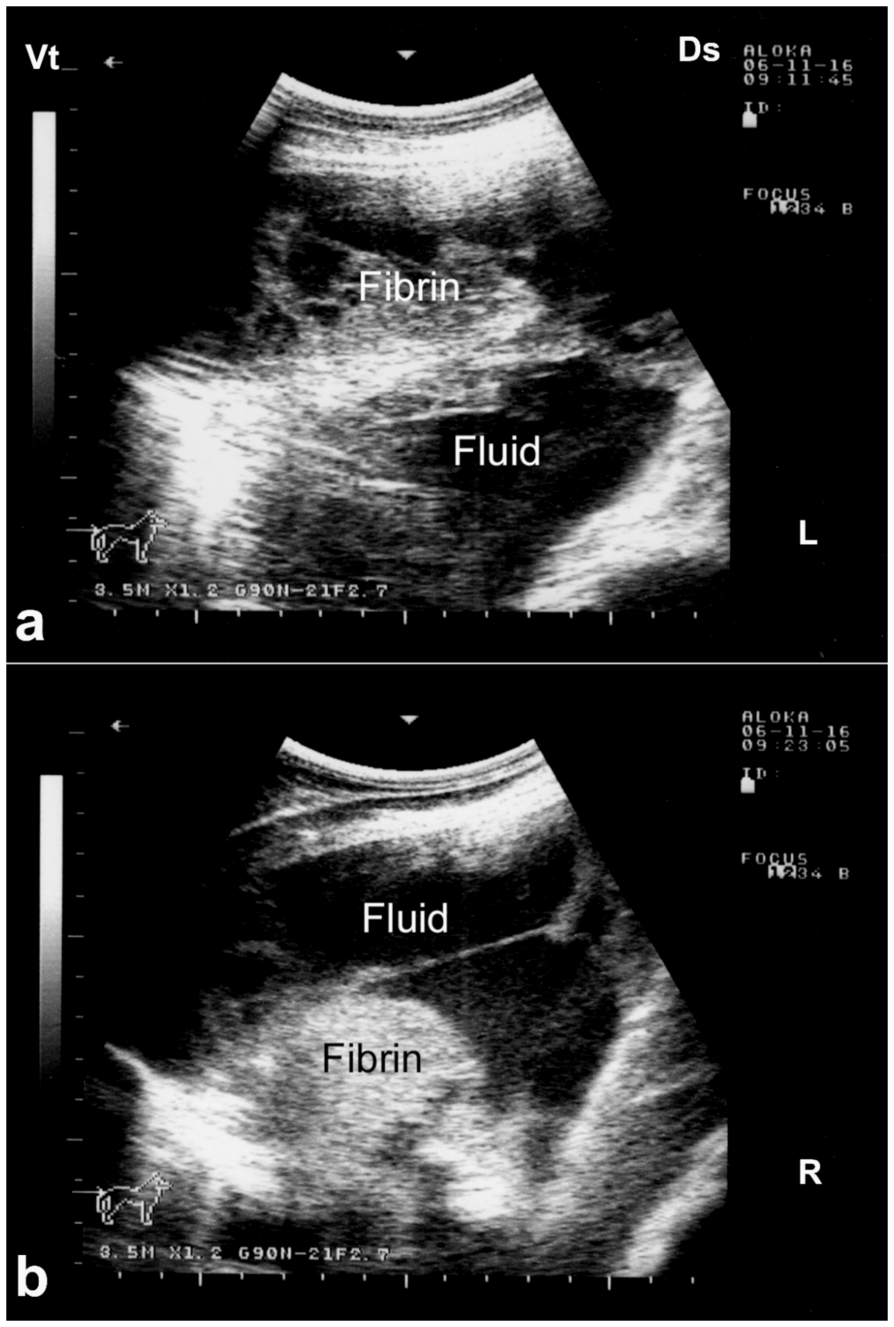
Ultrasonographic findings of bilateral fibrinous pleuropneumonia in a dromedary camel calf. **(a)** Left thoracic cavity; **(b)** Right thoracic cavity. Both images demonstrate extensive, hyperechoic fibrin strands floating within anechoic to hypoechoic pleural effusion, consistent with severe fibrinous pleuritis. The heterogeneous echotexture of the pleural fluid and evidence of adjacent pulmonary consolidation suggest concurrent lung involvement [modified from Tharwat and Al-Sobayil (8)].

Ultrasonography plays a vital role in the evaluation of pleurisy and pleuropneumonia, particularly where clinical signs are ambiguous or overlap with other respiratory conditions. In pleuritic cases, ultrasound can detect pleural thickening, fibrinous adhesions, and comet-tail artifacts arising from inflamed pleural surfaces. When pleuropneumonia is present, additional sonographic findings may include hypoechoic areas of lung consolidation, hepatization of pulmonary tissue, and pleural effusion with echogenic debris or septations—hallmarks of exudative inflammation (8). Ultrasound not only enables differentiation between pleural and pulmonary involvement but also facilitates monitoring of disease progression, planning of therapeutic drainage procedures, and sampling for microbiological diagnosis (31, 32). Its utility in field conditions makes it an indispensable tool for timely and accurate assessment of pleuropulmonary diseases in camelids. Thoracic ultrasonography is a valuable tool for monitoring pleural and pulmonary changes. It facilitates the detection of pleural effusion and helps in assessing prognosis (7). Ultrasonographic findings may include anechoic fluid accompanied by fibrin networks between the right and left pleurae. In advanced cases, bilateral heterogeneous pleural effusions containing fibrin strands can be observed and confirmed by ultrasound-guided thoracocentesis (Figure 13). In more severe instances, significant amounts of heterogeneous fluid with extensive fibrin networks can be seen in both pleural sacs (Figure 14) (8).

### 5.2 Cardiac diseases

To provide clinicians with a practical and rapid reference, a summary table (Table 2) has been included that outlines cardiopulmonary conditions observed in dromedary camels. The table concisely presents the etiology, common clinical signs, and characteristic ultrasonographic findings for each condition, including pericardial effusions, endocarditis, pericarditis, myocardial degeneration, and cardiomyopathies. This synthesis facilitates easier recognition and differentiation of these uncommon disorders during ultrasonographic cardiopulmonary assessment, ultimately supporting improved diagnosis and management in clinical practice.

**Table 2:** Summary of cardiopulmonary conditions in dromedary camels, including etiology, clinical signs, and ultrasonographic findings.

Condition	Etiology	Clinical signs	Ultrasonographic findings
Pericardial effusion	Chronic debilitating diseases (trypanosomiasis, chronic parasitism, malnutrition, hypoproteinemia, right-sided heart failure, severe anemia)	Muffled heart sounds (with large fluid volume)	Anechoic fluid collection surrounding the heart
Endocarditis	Rare; bacterial infection; one reported case with vegetative mitral valve lesion	Progressive exercise intolerance, reluctance to move, mild incoordination, weakness, pansystolic murmur	Not specifically described; diagnosis mainly postmortem
Pericarditis	Bacterial/viral infections, trauma, neoplasia, immune-mediated disorders, secondary to septicemia	Tachycardia, muffled heart sounds, tachypnea	Pericardial effusion, ventricular wall changes suggestive of tamponade; pleural effusion may be present
Myocardial degeneration	Selenium and/or vitamin E deficiency (nutritional muscular dystrophy)	Sudden collapse, tachycardia, increased respiratory rate, dullness; calves may be recumbent and weak	Tachycardia, increased echogenicity of the myocardium
Calcified cardiomyopathy	Advanced stage of myocardial necrosis, possibly vitamin E deficiency	Sudden death	Not described ultrasonographically; diagnosed postmortem
Hypertrophic cardiomyopathy	Unknown; reported case with thickened ventricular walls and reduced lumen	Recurrent syncopal episodes triggered by exercise or at rest	Echocardiography: thickened left ventricular free wall and interventricular septum, small ventricular lumen

#### 5.2.1 Pericardial effusions

Hydropericardium refers to the excessive accumulation of transudate within the pericardial sac. This condition is commonly associated with chronic debilitating diseases such as trypanosomiasis, chronic parasitism, prolonged malnutrition, severe hypoproteinemia, right-sided heart failure, and severe anemia. When large volumes of fluid accumulate, heart sounds may become muffled on auscultation. Ultrasonographic examination typically reveals an anechoic fluid collection surrounding the heart. Electrocardiographic (ECG) findings may include decreased amplitude of the P, QRS, and T waves (20).

#### 5.2.2 Endocarditis

Endocarditis is considered relatively rare in camels. To date, only one case has been documented in the literature, involving an 11-year-old castrated male dromedary camel with a 3–4-month history of progressive exercise intolerance (61). Initial clinical signs included reluctance to move, mild incoordination, and weakness even with minimal exertion. On physical examination, the camel was in poor body condition, with pale mucous membranes, a rectal temperature of 37.2 °C, a respiratory rate of 10 breaths per minute, and a heart rate of 48 beats per minute. There were no signs of respiratory distress, edema, or other abnormalities. Cardiac auscultation revealed a grade III pansystolic murmur localized to the 4th intercostal space. One week later, the animal’s condition deteriorated significantly and it was humanely euthanized. Postmortem examination revealed a firm heart upon palpation. Upon opening the heart, mild left ventricular hypertrophy was observed. Additionally, vegetative endocarditis of the mitral valve was identified. The lesion appeared as a hard, whitish, proliferative mass (61).

#### 5.2.3 Pericarditis

Pericarditis, or inflammation of the pericardium, is a relatively uncommon condition in camels. It may result from bacterial or viral infections, trauma, neoplasia, or immune-mediated disorders. In some cases, pericarditis develops as a secondary response to septicemic diseases. One reported case of traumatic pericarditis in a camel was documented by Hegazy et al. (62). Clinical signs include tachycardia, muffled heart sounds, and tachypnea are typical findings. Thoracic radiography shows diffuse thoracic opacity, dorsal deviation of the trachea, and absence of a clearly defined cardiac silhouette. Echocardiography reveals pericardial effusion, with changes in the ventricular walls suggestive of cardiac tamponade. Pleural effusion may also be present. Pericardiocentesis usually confirms the diagnosis and allows for analysis of pericardial fluid (20).

#### 5.2.4 Myocardial degeneration

**Figure 15:**
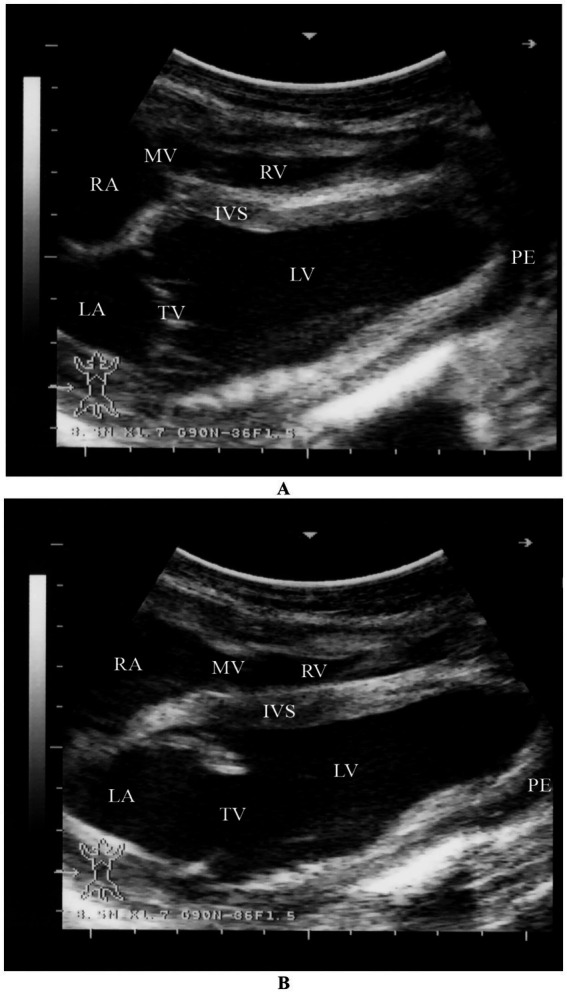
Two-dimensional echocardiographic assessment of a dromedary camel calf diagnosed with selenium and/or vitamin E deficiency (nutritional cardiomyopathy). **(A)** Right parasternal long-axis view during systole and **(B)** diastole demonstrate altered cardiac chamber dimensions. Quantitative measurements include right ventricular (RV), left ventricular (LV), right atrial (RA), and left atrial (LA) internal diameters. The interventricular septum (IVS) appears hypokinetic, and both tricuspid (TV) and mitral (MV) valves are identified for structural and functional assessment. These echocardiographic alterations, including chamber dilation and septal motion abnormalities, support the diagnosis of myocardial dysfunction secondary to nutritional cardiomyopathy [modified from Tharwat (32)].

Myocardial degeneration, also known as nutritional muscular dystrophy or white muscle disease, is a metabolic disorder caused by selenium and/or vitamin E deficiency. It can lead to necrotic myocarditis with heart failure or myocardial fibrosis, reducing cardiac efficiency. The disease is commonly seen in intensively managed camels and rapidly growing camel calves. It occurs in two clinical forms: acute (cardiac) and sub-acute (musculoskeletal). The acute form of the disease primarily affects the heart muscle and is often fatal. Affected animals may collapse and die suddenly following exertion, with no prior warning signs. Clinical signs include sudden onset of dullness, tachycardia, and increased respiratory rate. Camel calves are often found in lateral recumbency and may be unable to rise to sternal recumbency, even with assistance. Ultrasonography: Shows tachycardia and increased echogenicity of the myocardium (32) (Figure 15).

#### 5.2.5 Calcified and hypertrophic cardiomyopathy

Extensive myocardial degeneration and necrosis with fine dystrophic calcification of damaged cardiac muscle fibers have been reported in two dromedary camels that died suddenly in zoological gardens (63). Although the precise cause of cardiac muscle calcification remains unknown, it is generally considered an advanced stage in the process of myofiber necrosis. A deficiency of vitamin E in the diet was identified as the most likely underlying factor in these cases. To address this inconclusive etiology, future investigations should focus on evaluating trace nutrient levels—particularly selenium and/or vitamin E deficiency—in both healthy and affected camels. Experimental studies correlating serum antioxidant profiles with myocardial histopathology could offer valuable insights into the pathogenesis of calcific myocardial lesions. Hypertrophic cardiomyopathy has been reported in a 9-year-old castrated dromedary camel. The animal presented with a six-month history of recurrent episodes of syncope, initially occurring after moderate exercise and later even at rest. The syncopal episodes were sudden in onset and lasted between 30 to 45 s. Based on clinical evaluation, electrocardiography, and echocardiography, a tentative diagnosis of hypertrophic cardiomyopathy was made. At necropsy, the heart appeared globose in shape and was firm on palpation. Cross-sectional examination revealed marked thickening of the left ventricular free wall and the interventricular septum, with a correspondingly small left ventricular lumen (9). Unfortunately, a thorough review of the veterinary literature revealed no other published data regarding the diagnosis of cardiac diseases in dromedary camels using ultrasonography. This highlights the need for future prospective studies using echocardiography as a primary diagnostic tool for cardiac conditions in camels, particularly in animals with clinical signs suggestive of cardiomyopathy.

## 6 Comparison of ultrasonography with other diagnostic modalities

Ultrasonography offers key benefits over other imaging methods for evaluating camelid cardiopulmonary health, especially in field settings. Unlike auscultation, ultrasound directly visualizes conditions like pleural effusion and pericardial fluid that can be missed due to camels’ thick chest walls and stoic behavior (8). Radiography is limited by the camel’s size and positioning challenges, while ECG detects rhythm issues but lacks structural detail (64, 65). Advanced imaging like CT and MRI provide detailed anatomy but are costly, immobile, and require anesthesia, making them impractical for large animals (66). Ultrasound is portable, non-invasive, cost-effective, and suitable for real-time cardiac and lung assessment in standing camels with minimal restraint. It excels at detecting pleural and pericardial fluids, lung consolidation, and thoracic masses, and Doppler imaging assesses cardiac blood flow and valve function (31, 32).

Advanced ultrasound modalities, such as Doppler echocardiography, M-mode, and tissue Doppler imaging, provide enhanced functional assessment of cardiac performance, including real-time evaluation of ventricular wall motion, ejection fraction, and valvular regurgitation (31). These techniques allow for more precise hemodynamic evaluation, especially in cases of suspected heart failure or cardiomyopathies. Furthermore, lung ultrasound scoring systems and B-line quantification, adapted from human and small animal medicine, are being explored for their potential in grading pulmonary edema and monitoring respiratory diseases in camels (32).

However, its effectiveness is limited by lung air interference, operator skill, and reduced image quality in large animals (18). Although it cannot fully replace CT or MRI for deep thoracic imaging, ultrasound’s practicality makes it indispensable, especially when combined with other diagnostics. Used alongside auscultation and ECG, ultrasonography enhances diagnostic accuracy by clarifying ambiguous clinical signs and correlating electrical and mechanical heart function. It guides procedures like thoracocentesis and monitors disease progression or treatment response over time, improving safety and clinical decisions (14, 65, 66). In remote or resource-limited regions, ultrasound’s affordability, portability, and ease of use make it ideal for camel health management. It requires no sedation or radiation, enabling repeated evaluations with minimal cost and infrastructure. These features, along with relatively low training demands, position ultrasonography as a vital tool in camelid cardiopulmonary care, bridging gaps where advanced imaging is unavailable (18, 31).

## 7 Future perspectives and recommendations

To enhance the clinical utility of ultrasonography in camel cardiopulmonary diagnostics, several strategic developments are essential. First, establishing standardized imaging protocols tailored to the dromedary’s unique thoracic anatomy is crucial for consistency across practitioners. There is also a need for comprehensive, species-specific reference databases encompassing normal echocardiographic and pulmonary parameters across age, breed, and sex variations. Investment in capacity-building initiatives and structured training programs is important, especially in regions where camels are economically vital but advanced imaging remains limited. Integration with emerging technologies—such as artificial intelligence for automated interpretation and mobile-based telemedicine platforms—offers potential for expanding diagnostic reach and standardization in field conditions. Advanced ultrasonographic techniques such as contrast-enhanced ultrasound and Doppler imaging present promising avenues for improving assessment. Contrast-enhanced ultrasound can enhance visualization of myocardial and vascular structures, while Doppler provides insights into cardiac function and blood flow dynamics. Future research should focus on establishing reference values, validating these tools in large cohorts, evaluating prognostic relevance, and exploring multimodal approaches combining ultrasound with biomarkers, ECG, or molecular diagnostics for more accurate disease profiling.

## 8 Conclusion

This review highlights the expanding utility of ultrasonography as a non-invasive, accessible, and reliable modality for comprehensive cardiopulmonary assessment in dromedary camels. Key findings demonstrate that tailored ultrasound protocols can effectively delineate normal anatomical features and detect a broad spectrum of cardiac and pulmonary pathologies, which are often challenging to diagnose with traditional methods alone. The integration of ultrasonography into routine health management promises to improve early disease detection, guide therapeutic interventions, and ultimately enhance animal welfare and productivity. Nevertheless, the technique’s operator dependence and limited species-specific normative data underscore the urgent need for standardized imaging protocols and training programs. For veterinarians, adopting structured ultrasonographic approaches can augment clinical accuracy and decision-making in field and hospital settings. Meanwhile, researchers are encouraged to focus on validating reference values, refining imaging techniques, and exploring novel applications such as contrast-enhanced and Doppler ultrasonography to further elucidate camel cardiopulmonary physiology and pathology. Collectively, these advancements position ultrasonography as an indispensable tool in the evolving landscape of camelid veterinary medicine.
